# Integration of global metabolomics and lipidomics approaches reveals the molecular mechanisms and the potential biomarkers for postoperative recurrence in early-stage cholangiocarcinoma

**DOI:** 10.1186/s40170-021-00266-5

**Published:** 2021-08-04

**Authors:** Sureerat Padthaisong, Jutarop Phetcharaburanin, Poramate Klanrit, Jia V. Li, Nisana Namwat, Narong Khuntikeo, Attapol Titapun, Apiwat Jarearnrat, Arporn Wangwiwatsin, Panupong Mahalapbutr, Watcharin Loilome

**Affiliations:** 1 Department of Biochemistry, Faculty of Medicine, Khon Kaen University, 123 Mittraparp Road, Muang District, Khon Kaen, 40002 Thailand; 2 Cholangiocarcinoma Screening and Care Program (CASCAP), Khon Kaen University, Khon Kaen, 40002 Thailand; 3 Cholangiocarcinoma Research Institute, Faculty of Medicine, Khon Kaen University, Khon Kaen, 40002 Thailand; 4 Department of Surgery, Faculty of Medicine, Khon Kaen University, Khon Kaen, 40002 Thailand; 5 Department of Metabolism, Digestion and Reproduction, Faculty of Medicine, Imperial College London, London, SW7 2AZ UK

**Keywords:** Cholangiocarcinoma, Cancer recurrence, Metabolomics, Lipidomics, Biomarker

## Abstract

**Background:**

Cholangiocarcioma (CCA) treatment is challenging because most of the patients are diagnosed when the disease is advanced, and cancer recurrence is the main problem after treatment, leading to low survival rates. Therefore, our understanding of the mechanism underlying CCA recurrence is essential in order to prevent CCA recurrence and improve patient outcomes.

**Methods:**

We performed ^1^H-NMR and UPLC-MS-based metabolomics on the CCA serum. The differential metabolites were further analyzed using pathway analysis and potential biomarker identification.

**Results:**

At an early stage, the metabolites involved in energy metabolisms, such as pyruvate metabolism, and the TCA cycle, are downregulated, while most lipids, including TGs, PCs, PEs, and PAs, are upregulated in recurrence patients. This metabolic feature has been described in cancer stem-like cell (CSC) metabolism. In addition, the CSC markers CD44v6 and CD44v8-10 are associated with CD36 (a protein involved in lipid uptake) as well as with recurrence-free survival. We also found that citrate, sarcosine, succinate, creatine, creatinine and pyruvate, and TGs have good predictive values for CCA recurrence.

**Conclusion:**

Our study demonstrates the possible molecular mechanisms underlying CCA recurrence, and these may associate with the existence of CSCs. The metabolic change involved in the recurrence pathway might be used to determine biomarkers for predicting CCA recurrence.

**Supplementary Information:**

The online version contains supplementary material available at 10.1186/s40170-021-00266-5.

## Background

Cholangiocarcinoma (CCA), also known as bile duct cancer, is a malignant tumor that originates from the bile duct epithelium [1]. It has the highest incidence in Northeast Thailand where there is also a high incidence of *Opisthorchis viverrini* (OV) infection, the major risk factor of CCA development in this region [2]. Because of this high risk of OV infection, more than 1000 people are diagnosed with CCA every year at Srinagarind Hospital, Khon Kaen University [3]. CCA is thus a serious major health problem for people in this area. Surgery is the only curative treatment for CCA patients, and patient outcome is much improved when all residual tumor has been removed [4]. However, a high recurrence rate has been reported in CCA patients after surgery despite complete resection of the tumor, leading to an extremely poor prognosis [5, 6]. There is a substantially lower survival rate in patients with recurrence compared to those with non-recurrence (median overall survival 35.7 vs. 19.3 months) [5]. Recently, we reported the association of CSCs and recurrence with the overexpression of putative cancer stem-like cell (CSC) markers. These have the potential to predict CCA recurrence [7]. However, the mechanism involved in CCA recurrence is not well understood. Therefore, the study of the mechanism underlying CCA recurrence is still essential for managing disease and improving patient outcomes.

Reprogramming energy metabolism is defined as a hallmark of cancer development as it is required to balance energy production in order to support cancer survival and growth [8]. In cancer cells, oxygen is present, the glucose uptake rate is significantly increased, and lactate is produced [9]. This is the Warburg effect which was first noted in 1924 by Otto Warburg. Besides this, the alteration of other metabolic pathways, such as the lipogenic and amino acid metabolisms have also been reported to be involved in tumor progression [10, 11]. Therefore, metabolomics focuses on the analysis of low molecular weight compounds (metabolites) in biological samples, which become a powerful approach to uncover the mechanisms of many diseases including various types of cancer. In particular, it is widely used for biomarker discovery [12]. Untargeted/global metabolomics refers to the global detection of low molecular weight compounds in biological samples, while targeted metabolomics is the detection of defined groups of metabolites [13]. Apart from metabolomics, lipidomics is also important because lipids are biomolecules that have been reported to be involved in cancer progression [14]. Therefore, the study of both global metabolomics and lipidomics might be beneficial in providing comprehensive information on cancer metabolism and also cancer biomarker discovery.

Nowadays, metabolic biomarkers can be used in many clinical applications for patient assessment, including diagnosis and prognosis [15–18] as well as the identification of relevant biomarkers. The alteration of metabolites shows the potential to discriminate healthy controls from a patient with pancreatic adenocarcinoma with high efficacy [19]. In addition, there is evidence that serum/plasma metabolomics benefit cancer diagnosis [20–22] and the prognosis of cancer recurrence [23–25].

In this study, global metabolomics and lipidomics were used to compare CCA serum from patients with and without recurrence using ^1^H-nuclear magnetic resonance (^1^H-NMR) and ultra-performance liquid chromatography-mass spectrometry (UPLC-MS). The differential metabolites between recurrence and non-recurrence were used for pathway analysis to explore the mechanism underlying CCA recurrence. We found that patients with recurrence have lower levels of metabolites involved in mitochondrial respiration as well as higher levels of lipids compared with non-recurrence patients. There is considerable evidence suggesting that a low activity of mitochondrial respiration, as well as the induction of lipid uptake, is associated with the existence of CSCs [26] and has been reported in various cancer types including hepatocellular carcinoma (HCC) [27], melanoma [28], leukemia [29], and glioblastoma [30]. Interestingly, this metabolic feature is associated with CSCs which is an important factor for cancer recurrence. Therefore, we hypothesized that the alteration of metabolites in recurrence patients may be associated with the existence of CSCs, which lead to a higher risk of recurrence. To answer this hypothesis, the expression levels of putative CSC markers (CD44, CD44 variant 6, CD44 variant 8–10, and EpCAM), enzymes involved in lipid metabolism including CD36 (involved in lipid uptake), ATP citrate lyase (involved in lipid synthesis), and SCD1 (involved in lipid desaturation) were investigated. Moreover, the differential metabolites were further investigated for their prognostic efficacy for CCA recurrence and recurrence-free survival in order to identify potential biomarkers for CCA recurrence.

## Methods

### Patient sampling and follow-up

This was a retrospective study on OV-associated cholangiocarcinoma (CCA) patients who underwent surgery at Srinagarind Hospital, Khon Kaen University, Khon Kaen, Thailand, between 2007 and 2016. Pre-operative blood samples were collected from CCA patients and allowed to clot at room temperature before being centrifuged at 1000 g at 4°C for 10 min. Then, the serum was carefully collected into 1.5 mL tubes and stored at −80°C until analysis. CCA tissues were obtained from patients after surgery and kept in the Biobank of the Cholangiocarcinoma Research Institute. The patients were excluded if they received either radiotherapy or chemotherapy before surgery.

Patients were followed up every 3 months in the first year after surgery, then every 6 months thereafter. Computed tomography (CT)/magnetic resonance imaging (MRI) was performed to confirm postoperative recurrence in patients who have symptoms or signs of cancer recurrence. Recurrence-free survival (RFS) was measured from the date of surgery to recurrence or until the last follow-up in patients without recurrence. All subjects gave their informed consent for inclusion before they participated in the study. The study was conducted in accordance with the Declaration of Helsinki, and the study was approved by the Human Research Ethics Committee, Khon Kaen University, Thailand (HE611412).

### Sample preparation and acquisition for ^1^H-NMR spectroscopy

Prior to the metabolomics analysis, the frozen serum samples were defrosted at 4°C and mixed. Then, the samples were centrifuged and 300 μl of supernatant was gently mixed with 300 μl of serum buffer (0.075 M Na_2_HPO_4_ pH 7.4 in D_2_O_,_ 4.6 mM TSP, 0.004% NaN_3_). This was followed by centrifugation at 10,000 g, 4°C for 10 min. The mixed samples, 550 μl, were transferred into 5 mm NMR tubes (DWK Life Sciences, Germany). These were kept at 4°C until analysis.

^1^H-NMR spectra were acquired at 298 K using an NMR spectrometer at 400 MHz (Bruker, USA). The Carr-Purcell-Meiboom-Gill (CPMG) pulse sequence was employed to obtain spectra (recycle delay-90°-t1-90°-tm-90°-acquisition) in 64 scans.

### NMR spectral processing and statistical analyses

Data processing was performed using an in-house MATLAB script. Phase and baseline correction were performed in all NMR spectra, and the TSP peak was set as 0 ppm. After peak alignment, the water region was excluded (δ 4.2–δ 5.2). Pseudo-two-dimensional spectra were drawn in order to identify all metabolites by statistical total correlation spectroscopy (STOCSY), which confirmed the correlation of each resonance. Additionally, the resonances were searched against the Human Metabolome Database (HMDB), the ChenomxNMR Suite, and available literature. The integral area under the peak was obtained using the in-house MATLAB script. The concentration of peak of interest was presented by comparison with TSP that added was as an internal standard. The concentrations of interesting peaks were represented as median with interquartile range (IQR).

### Sample preparation and UPLC-MS analysis

The sample preparation and UPLC-MS analysis were performed as previously published [31, 32]. In brief, the frozen serum samples were sorted to the set of 80 in a rack. Then, the samples were defrosted at 4°C overnight and transferred into 96-deep-well polypropylene plates (2 mL, Eppendorf). The plates were sealed and centrifuged at 3486 g at 4°C for 10 min. After centrifugation, any solid debris was removed using a clean pipette tip. The supernatant (50 μl) was aliquoted into individual 96-well polypropylene plates, and four parts of isopropanol were added for protein precipitation. The 96-well polypropylene plates were sealed and then mixed at 1400 rpm at 4°C for 2 h. After mixing, the plates were centrifuged at 3486 g at 4°C for 10 min. 125 μL of supernatant was aspirated into a new 96-well polypropylene plate. The supernatant of each sample was also pooled to create the study reference (SR) sample in order to perform quality control (QC), which was performed throughout the analysis in every 10 study samples. In addition, SR samples were diluted through seven dilution series and acquired at the beginning and end of the run.

The prepared samples were examined using reversed-phase ultra-performance liquid chromatography (RP-UPLC). A 2.1×100 mm BEH C8 column (Waters Corp., UK) was used for analysis, and the column temperature was set at 55°C. Mobile phase A was a mixture of water, acetonitrile (ACN), isopropanol (IPA) in the proportion of 50:25:25 with 5 mM ammonium acetate, 0.05% acetic acid, and 20μM phosphoric acid. Solvent B was a mixture of ACN, IPA in the proportion of 50:50 with 5 mM ammonium acetate, and 0.05% acetic acid. The RP-UPLC was coupled with Xevo G2-S QTOF MS (Waters Corp., UK) via a Z-spray electrospray ionization (ESI) source for lipidomics analysis. The samples were acquired in both positive and negative ion modes in order to create the result in both positive and negative datasets, respectively.

### LC/MS data processing and statistical analyses

After data acquisition, XCMS was used for feature extraction [33]. In addition, the potential run-order effect elimination and feature filtering were performed using in-house and open-source scripts [34]. In order to gain only the features with high accuracy and high precision, features with the coefficient of variance (CV) in SR samples less than 20% and features correlated to SR dilution which showed a Pearson correlation coefficient greater than 0.8 were retained. After that, the data matrix was normalized using median fold change normalization. The data file was subjected to multivariate analysis using SIMCA 14 software (Umetricas, Sweden). After orthogonal partial least square discriminate analysis (OPLS-DA) was applied, the variables with relevance to the discrimination between recurrence (R) and non-recurrence (NR) based on a p(corr) cut-off of |0.5|together with variables important in the projection (VIP) score above 1.0 were selected. The data was analyzed using the Mann-Whitney *U* test in MetaboAnalyst 4.0 software; variables with a false discovery rate (FDR) adjusted *p* value less than 0.05 were selected for further analysis

Subsequently, the significant features were identified using m/z by matching with online databases (Metline and HMDB). Then, the structure of the lipids was investigated using MS/MS fragmentation patterns. The level of the assignment was grouped based on the previously published criteria [35]: (1) m/z matched to database, (2) m/z matched to database and MS/MS fragment matched to in silico fragmentation pattern, (3) MS/MS fragment matched to database or literature review, (4) retention time matched to standard compound, and (5) MS/MS fragment matched to standard compound.

### Antibodies

The antibodies used in this study were mouse monoclonal anti-CD44 (1:100; #ab516728), mouse monoclonal anti-CD44v6 (1:50; #ab78960), rabbit polyclonal anti-EpCAM (1:100; #ab71916), rabbit monoclonal anti-CD36 (1:25; #ab133625), rabbit monoclonal anti-ATP citrate lyase (1:200; #ab40793), rabbit monoclonal anti-SCD1 (1:100; #ab236868) and HRP-conjugated rabbit anti-rat (1:50; #ab6734) antibodies (Abcam, CA), and rat monoclonal anti-CD44v8-10 antibody (1:50; #LKG-M001) (Cosmo Bio, JP).

### Immunohistochemistry (IHC) and scoring

Two independent punctures from paraffin-embedded tissues of each patient were used to produce tissue microarrays (TMA). Tissue sections were de-paraffinized and rehydrated stepwise of xylene, 100%, 90%, 80%, and 70% ethanol, respectively. Microwave cooking was used for antigen retrieval with 10-mM sodium citrate; pH 6; and 0.05% Tween20 for CD36, CD44, and CD44v6, whereas in the Tris-EDTA, pH 9 was used for ATP-citrate lyase, SCD1, EpCAM, and CD44v8-10. Endogenous hydrogen peroxide activity and nonspecific binding were blocked with 0.3% hydrogen peroxide and 10% skim milk for 30 min. Primary antibody was added and incubated at room temperature for 1 h, then at 4°C overnight. After washing, secondary antibody (Dako EnVision) was added for 1 h, except for CD44v8-10. HRP conjugated anti-rat was added and left for 3 h. A 3,3-diaminobenzidine tetrahydrochloride (DAB) substrate kit (Vector Laboratories, Inc., Burlingame, CA) was used for signal development. Sections were then counterstained with Mayer’s hematoxylin. Dehydration was performed stepwise of 70%, 80%, 90%, and 100% ethanol and xylene, respectively, and mounted with Permount. Stained sections were viewed under a light microscope.

The IHC score of each patient was calculated as the average score from two independent punctures. Staining frequency and intensity were used for scoring. The percentage of positive cancer cells was defined as the frequency with 0%=negative, 1–25%=+1, 26–50%=+2, and>50%=+3. The intensity was scored as three levels, weak=1, moderate=2, and strong=3. The range of final scores was 0–9, determined by multiplying the intensity with the frequency. IHC score was calculated as a median value and used as a cut-off point. Patients were classified as low or high expression groups if the grading score was lower or equal to or higher than the median, respectively. For protein having a median value equal to zero, patients were classified into negative or positive expression groups if the grading score was equal to or higher than zero, respectively.

### Statistical analysis

The results from global metabolomics and lipidomics were analyzed using SPSS statistical package version 25 and SIMCA software 14 together with MetaboAnalyst 4.0 software. The differential metabolites were further analyzed using hierarchical clustering and correlation heatmap analysis, metabolic pathway analysis, and also the receiver operator characteristic (ROC) curve using MetaboAnalyst. For IHC results, the correlation between proteins was analyzed using correlation heatmap analysis, MetaboAnalyst. The association between metabolic levels, protein levels, and RFS was analyzed by Kaplan-Meier survival analysis and the log-rank test using SPSS. A *p* value less than 0.05 was considered as statistically significant.

## Results

### Patient characteristics and patient outcomes

A total 102 CCA patients were enrolled in this study, and we firstly analyzed the association of patient characteristics including age, sex, tumor site, histology type, and tumor staging (according to the 7th edition of the American Joint Committee on Cancer (AJCC) Staging Manual) with clinical outcomes, including recurrence-free survival (RFS) and overall survival (OS). Our results indicated that patient outcomes were mostly affected by the stage of cancer, including primary tumor (T stage) (RFS; *p* = 0.021, OS; *p* < 0.001), lymph node metastasis (N stage) (OS; *p* < 0.001), distant metastasis (M stage) (OS; *p* < 0.001), and TNM stage (RFS; *p* = 0.007, OS; *p* < 0.001) (Fig. S1 and S2). Serum metabolomics were thus studied according to cancer staging, including early stage (TNM stage 0-II) and late stage (TNM stages III–IV) to avoid the effect of T, N, M, and TNM stage on cancer recurrence.

Serum metabolomics were analyzed separately based on staging. Among all patients, 91 cases were included for global metabolomics, with 36 cases (39.6%) from early stage and 55 cases (60.4%) from late-stage CCA. For lipidomics, 101 cases were analyzed, with 42 cases (41.6%) from early stage and 59 cases (58.4%) from late-stage CCA. The other patient characteristics for global metabolomics and lipidomics are summarized in Table S1 and Table S2, respectively.

### Global metabolomics and lipidomics analysis of recurrence (R) and non-recurrence (NR) in CCA patients

**Fig. 1:**
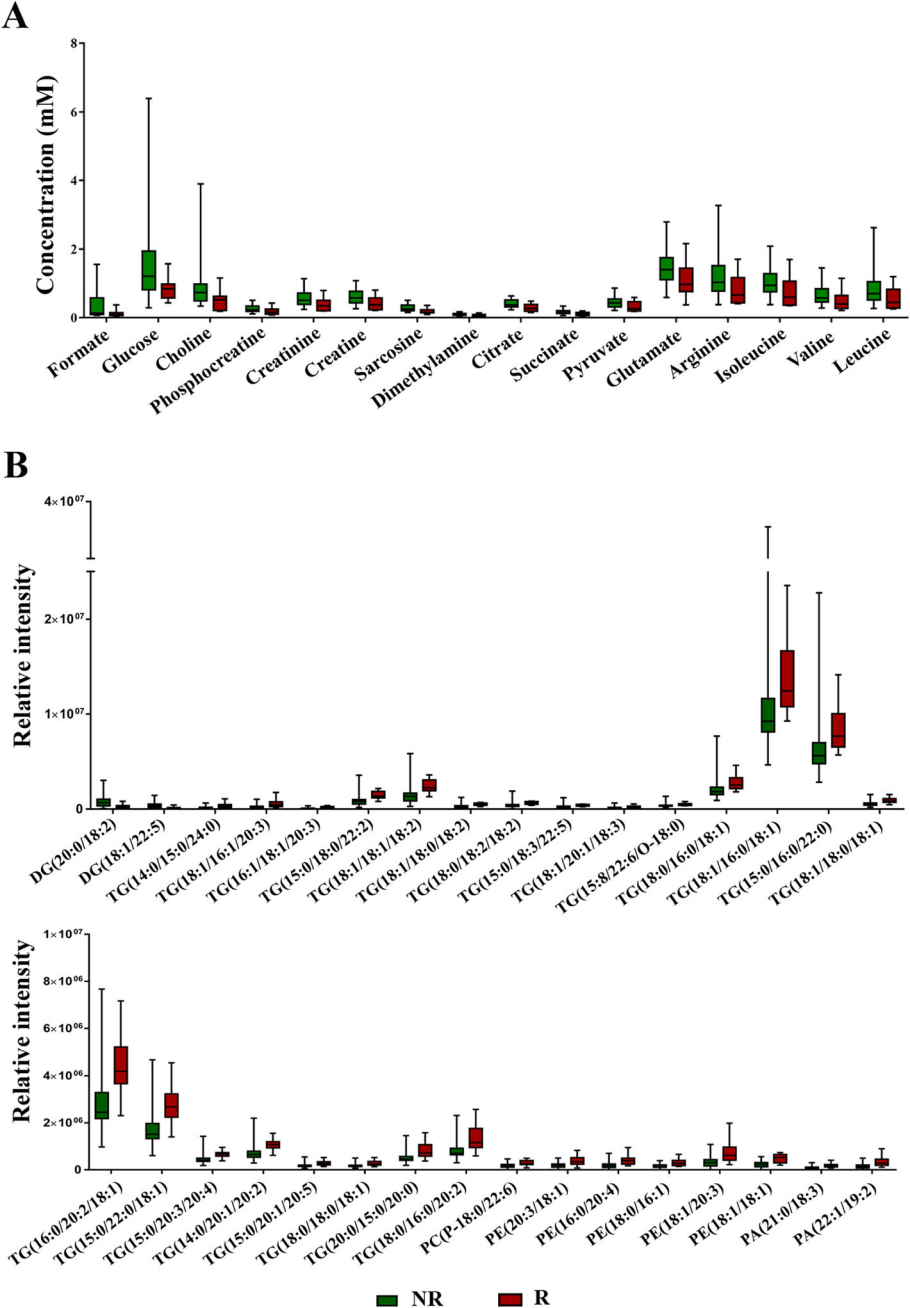
The Box and Whisker plot shows the different metabolic profiles between recurrence and non-recurrence patients. **A** The significant differential metabolites from global metabolomics. **B** The significant differential lipid species from lipidomics. NR non-recurrence, R recurrence, DG driacylglycerol, TG triacylglycerol, PC phosphatidylcholine, PA phosphatidic acid, PE phosphatidylethanolamine

To understand the mechanism underlying CCA recurrence, we performed global metabolomics and lipidomics using ^1^H-NMR and UPLC-MS, respectively. For global metabolomics, 36 cases from early stage (NR = 26, R = 10) and 55 cases from late stage (NR = 37, R = 18) were analyzed using ^1^H-NMR. A total 29 metabolites were identified from CCA serum, and the number of metabolites in the early and late stage did not differ. Among them, 16 metabolites including leucine, valine, isoleucine, arginine, glutamate, pyruvate, succinate, citrate, dimethylamine, sarcosine, creatine, creatinine, phosphorcreatine, choline, glucose, and formate showed significant differences between the recurrence and non-recurrence groups in early-stage patients (Table S3 and Fig. 1A), while no significant differences in metabolites were found in late-stage patients.

For lipidomics, the metabolic profiles of serum from CCA patients with and without recurrence were characterized by UPLC-MS in both positive and negative modes. A total of 42 cases were classified as early stage (NR = 12, R = 30) and 59 were classified as late stage (NR = 40, R = 19). In the early stage patients, 32 metabolites were significantly different between patients with and without recurrence, of which 26 lipid species were from the positive mode and 6 from the negative mode. The differential lipid species of patients with and without recurrence are shown in Table S4 and Fig. 1B: these include 2 driacylglycerols (DGs) and 22 triacylglycerols (TGs), 1 phosphatidylcholine (PCs), 5 phosphatidylethanolamines (PEs), and 2 phosphatidic acids (PAs). On the other hand, there were no significant differences in metabolites between patients with and without recurrence in late-stage CCA. Therefore, only early-stage CCA samples were used for further analysis.

**Fig. 2:**
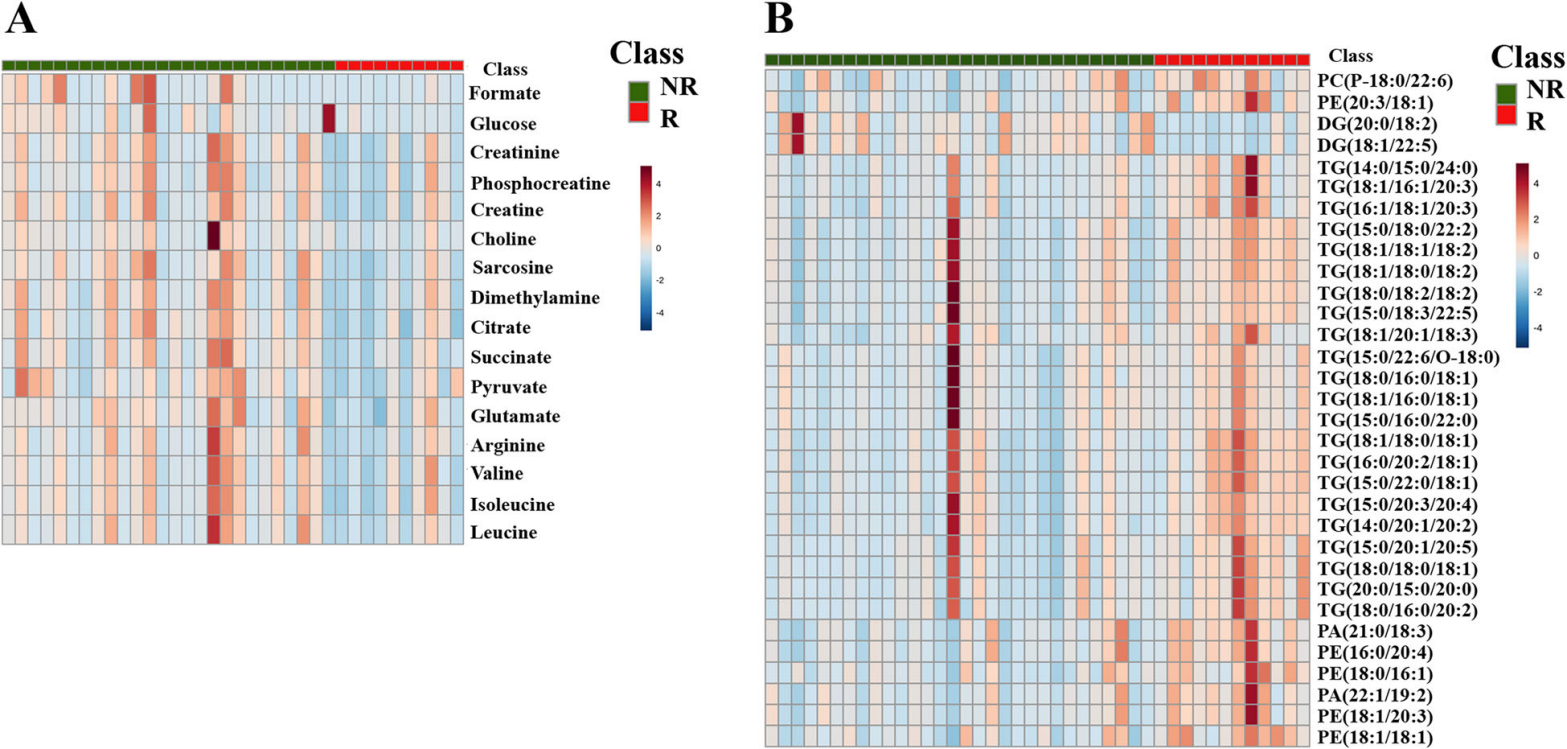
Heatmap analysis at the level of differential metabolites between recurrence and non-recurrence.**A** The result from global metabolomics. **B** The result from lipidomics. The row represents metabolites, and the column represents individual samples. The color bars on the top right of the heatmap indicate the level of metabolites with red and blue representing the highest and lowest levels, respectively. NR non-recurrence, R recurrence, DG driacylglycerol, TG triacylglycerol, PC phosphatidylcholine, PA phosphatidic acid, PE phosphatidylethanolamine

A heatmap analysis at the level of differential metabolites in each sample is shown in Fig. 2A, B. The levels of metabolites are indicated by the degree of color. The results from global metabolomics indicate that all metabolites were downregulated in recurrence patients compared with non-recurrence patients (Fig. 2A). For lipidomics, compared with non-recurrence patients, most of the lipid species were upregulated in recurrence patients, including TG, PC, PE, and PA, while the level of DG was downregulated in recurrence patients (Fig. 2B).

**Fig. 3:**
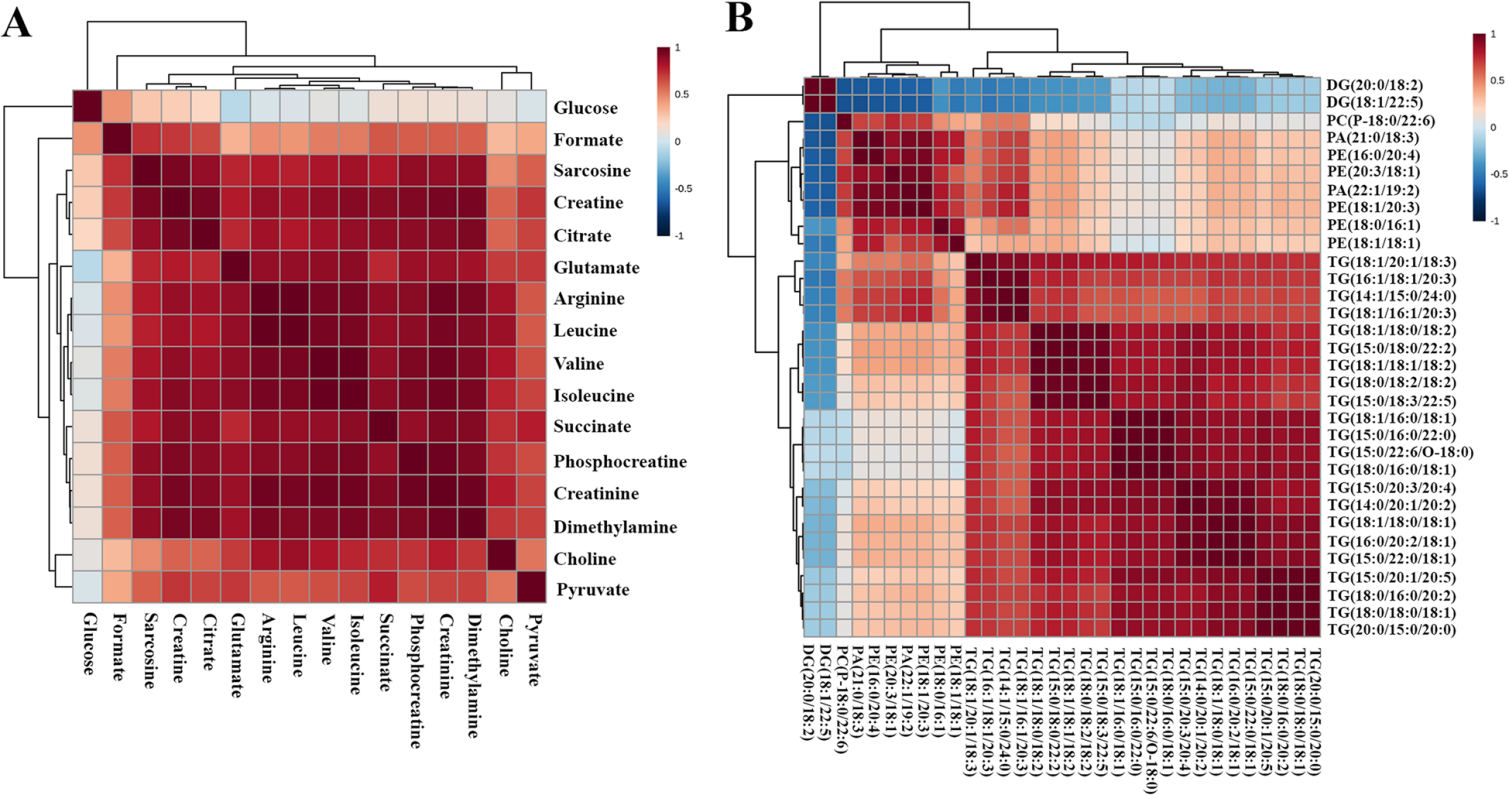
The correlation heatmap with a hierarchical clustering of all differential metabolites between recurrence and non-recurrence. **A** The result from global metabolomics.**B** The result from lipidomics. The magnitude of the correlation between the metabolites is shown with red representing a positive correlation and blue a negative correlation. DG driacylglycerol, TG triacylglycerol, PC phosphatidylcholine, PA phosphatidic acid, PE phosphatidylethanolamine

The correlation heatmap with a hierarchical clustering of all significant metabolites is shown in Fig. 3A, B. The magnitude of the correlation of metabolites is shown by color. Global metabolomics showed that most of the metabolites have a positive relationship to the others (Fig. 3A). The results from lipidomics showed that most of identified lipids have a good correlation to others (Fig. 3B).

### Pathway analysis of differential metabolites

**Fig. 4:**
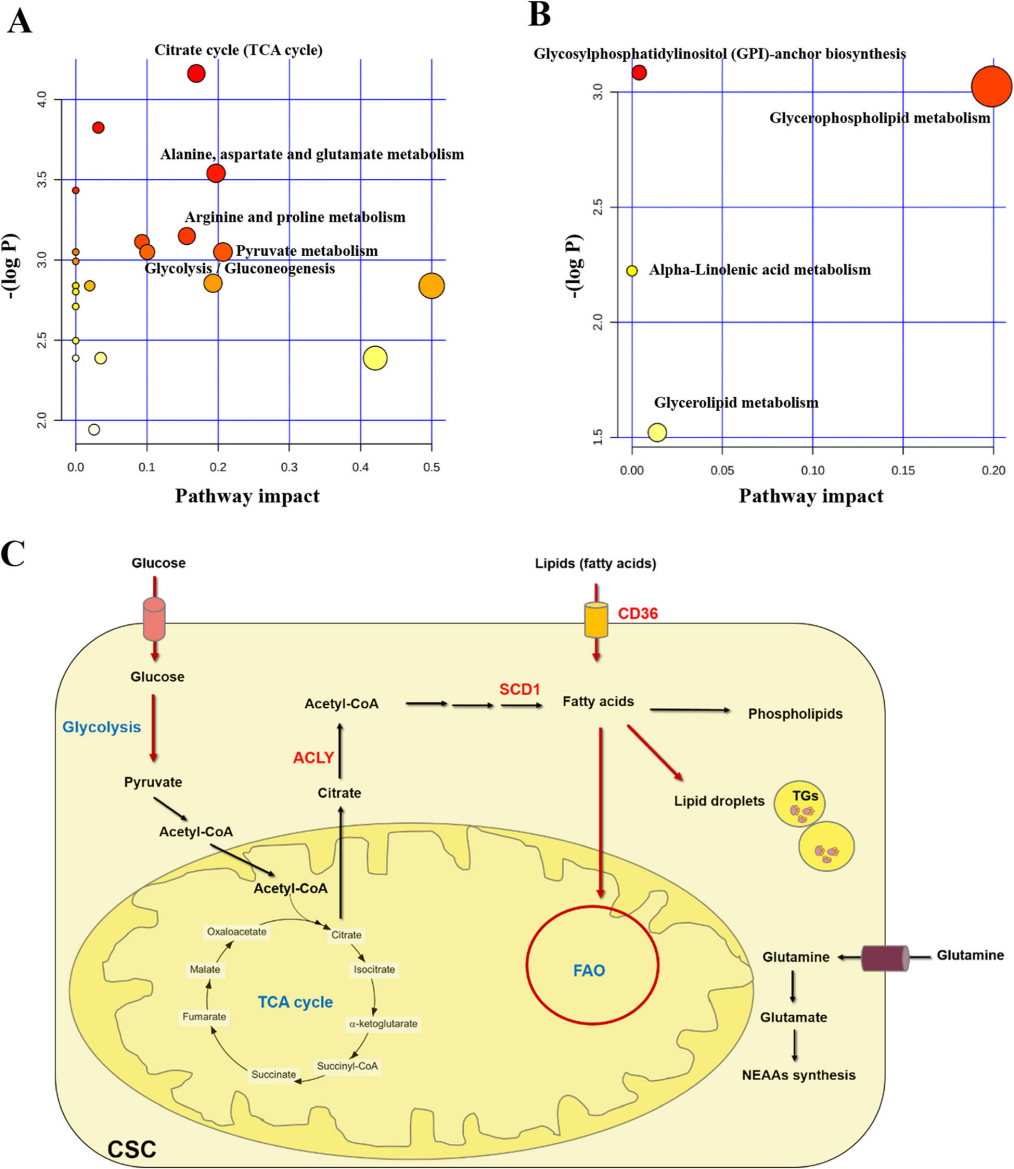
The summary of pathway analysis on differential metabolites between recurrence and non-recurrence, analyzed using MetaboAnalyst 4.0. **A** Metabolism pathway analysis from global metabolomics. **B** Metabolism pathway analysis from lipidomics. The color of the circle represents the *p* value, and the size of the circle represents the pathway impact. **C** The schematic diagram of metabolic pathways involved in CCA recurrence with red arrows indicating the most relevant pathway for recurrence. FAO fatty acid oxidation, CSC cancer stem cell, TG triacylglycerol, NEAAs non-essential amino acids, CD36 cluster of differentiation 36, ACLY ATP citrate lyase, SCD1 stearoyl-CoA desaturase-1

The metabolic pathway relevant to the differential metabolites between recurrence and non-recurrence in both global metabolomics and lipidomics were examined using pathway analysis by MetaboAnalyst 4.0. This analysis is based on pathway enrichment and topology analysis. The results from global metabolomics revealed that the differential metabolites were mostly involved in 7 pathways of which pyruvate metabolism, alanine-aspartate-glutamate metabolism, the citrate cycle (TCA cycle), arginine and proline metabolism, and glycolysis/gluconeogenesis were considered as the most relevant pathways involved in CCA recurrence according to their impact values (Fig. 4A and Table 1). In addition, pathway analysis on the differential lipid species demonstrated that glycerophospholipid metabolism, glycerolipid metabolism, and glycosylphosphatidylinositol (GPI)-anchor biosynthesis were the relevant pathways for CCA recurrence (Fig. 4B and Table 2). A schematic diagram of the metabolic networks involved in CCA recurrence is presented in Fig. 4C.

**Table 1:** Pathway analysis of differential metabolites from global metabolomics

Pathways	Hits	Raw ***p***	Holm adjust	FDR	Impact
Pyruvate metabolism	1	0.047	0.901	0.083	0.207
Alanine, aspartate and glutamate metabolism	4	0.029	0.668	0.083	0.197
Citrate cycle (TCA cycle)	3	0.016	0.389	0.083	0.169
Arginine and proline metabolism	5	0.043	0.901	0.083	0.156
Glycolysis/gluconeogenesis	1	0.047	0.901	0.083	0.100
Glycine, serine, and threonine metabolism	4	0.044	0.901	0.083	0.093
Glyoxylate and dicarboxylate metabolism	4	0.022	0.524	0.083	0.032
Propanoate metabolism	1	0.032	0.711	0.083	0
Cysteine and methionine metabolism	1	0.047	0.901	0.083	0
Tyrosine metabolism	1	0.047	0.901	0.083	0
Butanoate metabolism	2	0.050	0.901	0.083	0

*Hits* matched metabolites in the pathway, *raw p* original *p* value calculated from enrichment analysis, *Holm adjust* adjust *p* value from Bonferroni method, *FDR* false discovery rate, *Impact* pathway impact calculated from topology analysis

**Table 2:** Pathway analysis of differential metabolites from lipidomics

Pathways	Hits	Raw ***p***	Holm adjust	FDR	Impact
Glycerophospholipid metabolism	2	0.0009	0.005	0.003	0.199
Glycerolipid metabolism	1	0.0301	0.030	0.030	0.014
Glycosylphosphatidylinositol (GPI)-anchor biosynthesis	1	0.0008	0.005	0.003	0.004
Arachidonic acid metabolism	1	0.0060	0.024	0.007	0
Linoleic acid metabolism	1	0.0060	0.024	0.007	0
Alpha-Linolenic acid metabolism	1	0.0060	0.024	0.007	0

*Hits* matched metabolites in pathway, *Raw p* original *p* value calculated from enrichment analysis, *Holm adjust* adjust *p* value from Bonferroni method, *FDR* false discovery rate, *Impact* pathway impact calculated from topology analysis

### Evaluation of protein expression by immunohistochemistry (IHC)

**Fig. 5:**
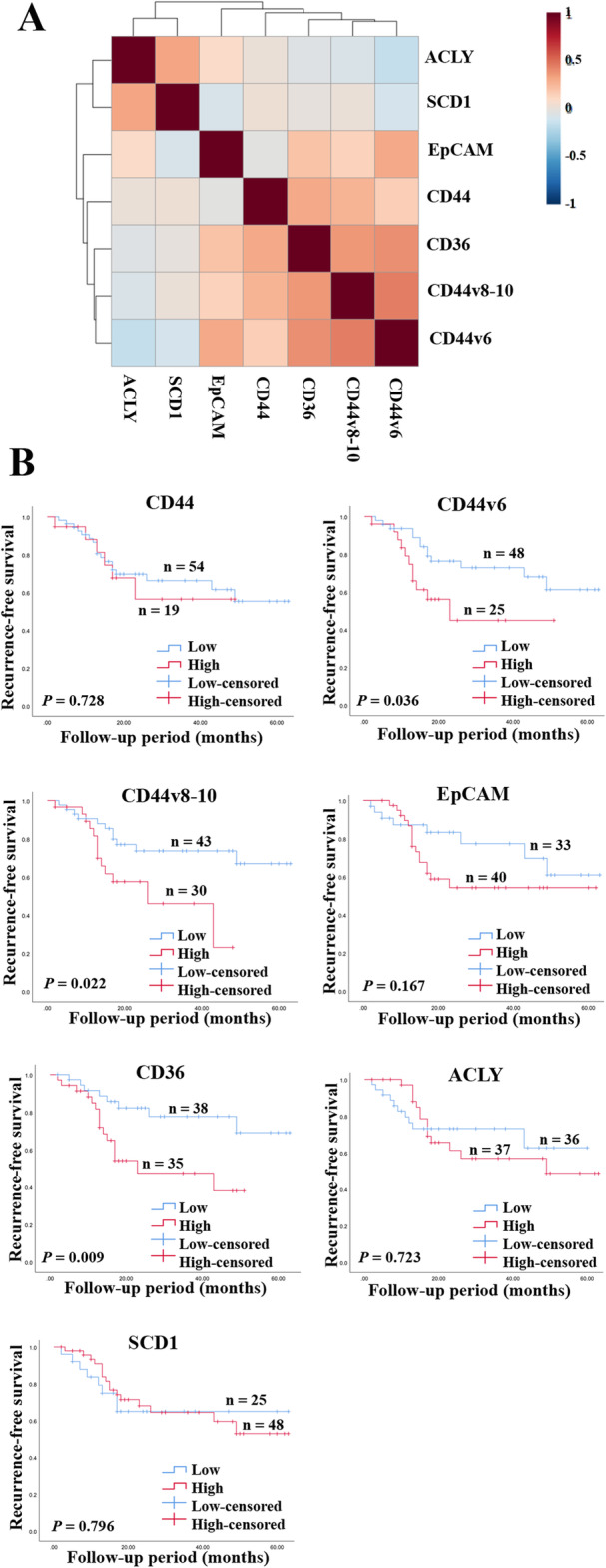
The correlation between CSC markers and proteins involved in lipid metabolism and their importance in the patient’s outcome. **A** The correlation heatmap with a hierarchical clustering of the levels of CSC markers and proteins involved in lipid metabolism. The magnitude of the correlation is shown by the colors with red representing a positive correlation and blue a negative correlation. **B** Kaplan-Meier curves representing the correlation between protein expression and recurrence. Low represents a low protein expression, high represents a high protein expression. CD cluster of differentiation, EpCAM epithelial cell adhesion molecule, ACLY ATP citrate lyase, SCD1 stearoyl-CoA desaturase-1. A *p* value lower than 0.05 was considered as a significant value

We explored the association between the alteration of metabolites and the existence of CSCs which may lead to CCA recurrence. The expression levels of putative CSC markers (CD44, CD44 variant 6, CD44 variant 8-10, and EpCAM), CD36, ATP citrate lyase, and SCD1 were examined. The association of these proteins in each pathway is shown in Fig. 4C. The results from IHC indicate that the expression level of CD36 was significantly associated with the expression level of the CSC markers, CD44, CD44v6, and CD44v8-10 (Fig. 5A and Table S5). Moreover, CCA patients with high levels of CD36, CD44v6, and CD44v8-10 have significantly shorter recurrence-free survival than those with low expressions (Fig. 5B).

### Predictive performance of biomarkers on recurrence

**Fig. 6:**
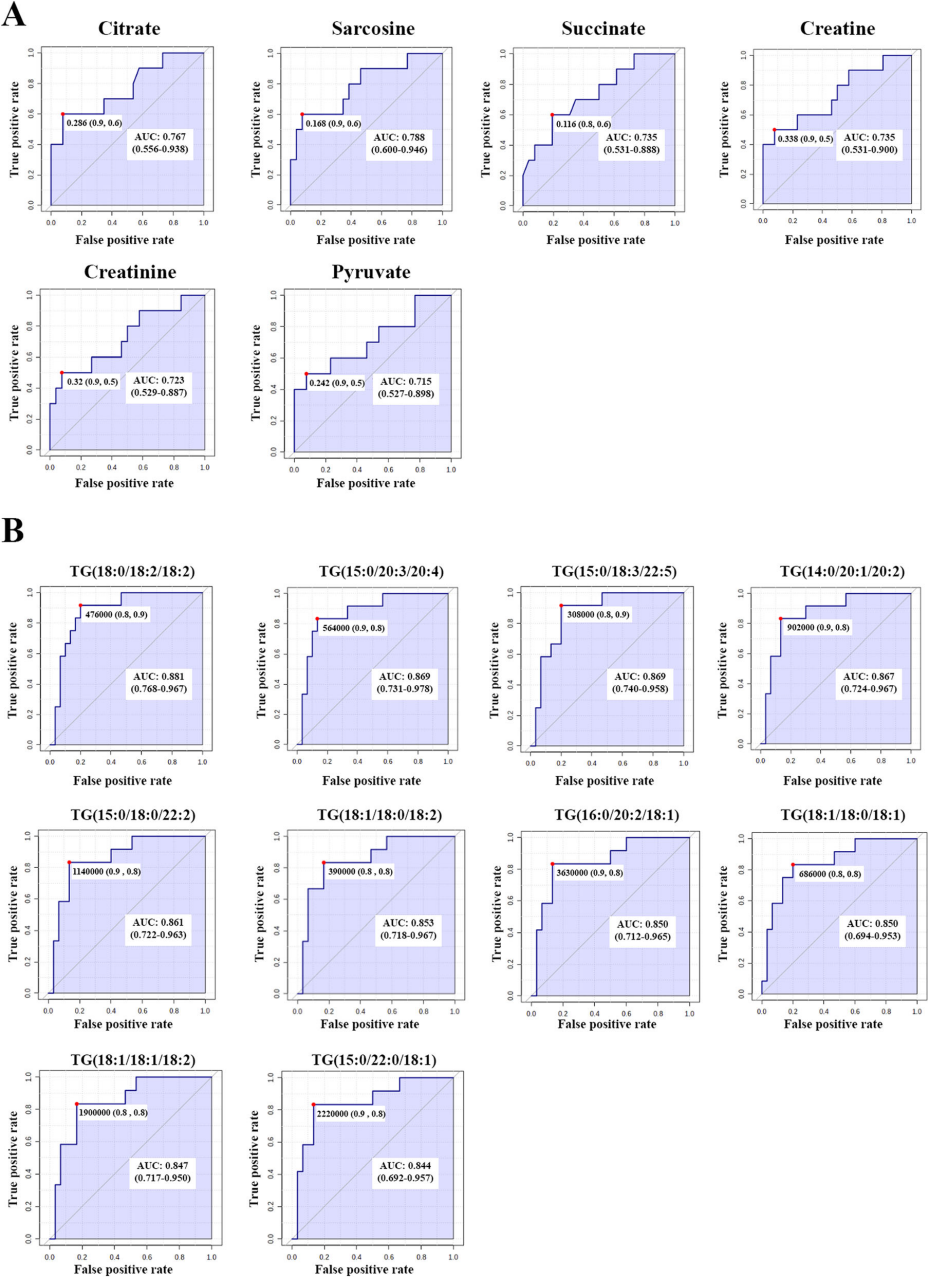
ROC curve analysis on differential metabolites between recurrence and non-recurrence. **A** The potential metabolic biomarkers from the global metabolomics result. **B** The potential metabolic biomarkers from the lipidomics result. The area under the curve (AUC), sensitivity, and specificity at the optimal cut-off derived by Youden’s index. TG triacylglycerol

We found alterations in the levels of metabolites associated with CCA recurrence. Thus, these metabolic changes may be used as biomarkers for CCA recurrence. In order to evaluate the predictive value of individual metabolites, we performed ROC analysis in all significant differential metabolites. The result from global metabolomics showed that 6 metabolites (citrate, sarcosine, succinate, creatine, creatinine, and pyruvate) have a predictive efficacy on CCA recurrence (*p* < 0.05) with the area under curve analysis (AUC) > 0.7 (Fig. 6A). For lipidomics, we found 28 lipid species that showed a predictive efficacy on CCA recurrence (*p* < 0.05). Among these 28 lipid species, the top 10 lipid species had the highest AUC (AUC > 0.8) (Fig. 6B).

### Kaplan-Meier analysis of metabolites on recurrence-free survival

**Fig. 7:**
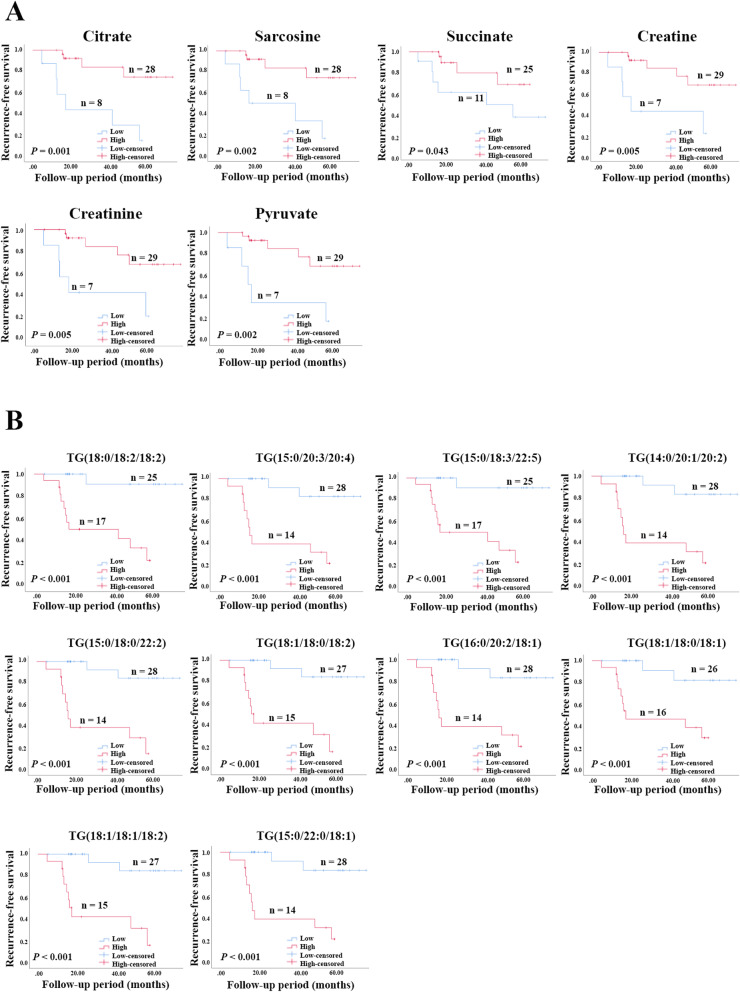
Kaplan-Meier curves represent the correlation between metabolic levels and recurrence. **A** The result of potential metabolic biomarkers from global metabolomics. **B** The result of potential metabolic biomarkers from lipidomics. Low represents the level of metabolite lower than the optimal cut-off, high represents the level of metabolite higher than or equal to the optimal cut-off. TG triacylglycerol. A *p* value lower than 0.05 was considered as a significantly value

In order to evaluate the prognostic performance of metabolites on a patient’s outcome, recurrence-free survival analysis was performed on the potential metabolite biomarkers. Based on Youden’s index (Youden’s index = sensitivity + specificity - 1), the max value was used as a cut-off for low and high levels. The results show that 6 metabolites from global metabolomics have a prognostic effect on CCA recurrence. It was revealed that patients with low levels of citrate, sarcosine, succinate, creatine, creatinine, and pyruvate have a significantly shorter recurrence-free survival than those with high levels (Fig. 7A). In addition, recurrence-free survival based on lipid biomarkers was also analyzed. The results demonstrated that patients with high levels of TGs have significantly lower recurrence-free survival compared with those patients with low levels (Fig. 7B).

## Discussion

Achieving long-term survival for CCA patients after treatment is the major challenge clinical because many patients develop recurrence after surgery, leading to the low survival rates [5, 36–38]. Therefore, the prevention of cancer recurrence is the major clinical focus following surgery. There is substantial evidence demonstrating the role of prognostic markers such as tumor size, tumor number, and metastasis status on CCA recurrence, and these have been suggested as prognostic markers for CCA recurrence [5, 6, 38, 39]. In this study, we also found that T and TNM stages have the potential as prognostic markers for recurrence-free survival. However, an understanding of the molecular mechanisms involved in CCA recurrence, as well as the identification of effective molecular biomarkers, is still needed to help manage the progression of cancer. Moreover, molecular biomarkers could be developed as a drug target for the prevention of CCA recurrence.

The mechanisms underlying cancer recurrence have been reported for many cancers. These are mostly associated with a subpopulation of cancer cells that are resistant to therapeutics and are called cancer stem-like cells (CSC) [40–42]. Our previous report found an association between CSC markers with CCA recurrence, suggesting that CCA recurrence may be associated with CSCs [7]. In addition, reprogramming of metabolism is known as the hallmark of cancer [43], and such metabolic changes have also been reported in CSCs [26]. Based on this background, we used high-throughput metabolomics technology integrating global metabolomics and lipidomics approaches to reveal the deferential metabolites on CCA patients with and without recurrence. This will benefit our understanding of the recurrence process and potentially identify effective biomarkers for CCA recurrence. From our study, we found that early-stage CCA patients with and without recurrence have different metabolic profiles, while this was not found in the late-stage group. This is consistent with our previous study which found that molecular biomarkers have the potential to predict CCA recurrence only in early-stage patients [7]. After pathway analysis was performed, the result from global metabolomics revealed that the differential metabolites between recurrence and non-recurrence are mostly involved in energy and amino acid metabolisms. We found that recurrence patients have a high activity of glycolysis and pyruvate metabolism, which are represented by low levels of glucose and pyruvate, together with a low activity of the TCA cycle represented by low levels of citrate and succinate. There have been evidences that the CSCs are involved in cancer recurrence and tend to use glycolysis instead of the TCA cycle which is coupled with oxidative phosphorylation (OXPHOS) to maintain stemness and survival as it can minimize reactive oxygen species (ROS) production and induce detoxification systems [27, 44, 45]. Thus, our result leads us to focus on the metabolism of CSCs which may lead to CCA recurrence.

Amino acids are the building blocks for protein synthesis. It has been reported that amino acids are the major biomass in proliferating mammalian cells [46]. Unlike other organisms, mammalian cells cannot synthesize all of the necessary amino acids, some of which, called essential amino acids (EAA) which must be acquired from the diet [47]. On the other hand, the synthesis of other nonessential amino acids (NEAA) is mostly associated with glutamine, which is further converted to glutamate via a deamination reaction modulated by the glutaminase (GLS) [48]. This leads to the biosynthesis of other NEAAs such as proline, aspartate, asparagine, and alanine [47]. In the proliferating state, the synthesis of macromolecules is needed for constructing new cells [49]. There is evidence that proliferating cells use more glutamate for NEAA synthesis, while quiescent cells show low levels of glutamine consumption, indicating low levels of NEAA synthesis [50]. Proliferating cells need more NEAA production to support biosynthesis. Unlike proliferating cells, slow-cycling or quiescent CSCs are known as the suppopulation of cancer cells with stem-like properties; an important property that leads to drug resistance [51]. In our study, we found differential metabolites mostly associated with NEAA metabolism pathways, which include alanine, aspartate and glutamate metabolism, and arginine and proline metabolisms. Moreover, recurrence patients also showed low levels of metabolites involved in these pathways. Therefore, our results suggest that recurrence patients may have a higher population of quiescent CSCs, leading to a higher risk of recurrence.

Besides the alteration in metabolism-related to glucose and amino acids, the alteration of lipid metabolism is also important for many cancers. In fact, lipids are biomolecules that are essential for many cellular processes, including cell proliferation, signal transduction, and energy storage [52]. Lipidomics thus becomes a powerful tool for studying lipid profiles in cancer. In ovarian cancer, the lipid profile could discriminate between patients with and without recurrence, and thirty-one lipid species could be used as potential biomarkers for tumor recurrence [17]. The association between lipids and recurrence was also found in prostate cancer, with a high level of serum triglycerides associated with a high risk of prostate cancer recurrence [53]. There is also evidence showing the association between lipid metabolism and cancer recurrence. Therefore, we further hypothesized that an alteration of lipid metabolism may also be associated with the existence of CSCs leading to CCA recurrence. In CSCs, the alteration of lipid metabolism is also important for maintaining survival and stemness. The fatty acid oxidation (FAO) pathway to produce energy from lipids plays an important role in energy production in CSCs [26]. FAO is elevated in breast cancer stem cell (BCSCs), which is required for self-renewal and chemoresistance [54]. In hepatocellular carcinoma (HCC), NANOG, a well-known stem cell marker, suppresses OXPHOS and mitochondrial ROS production as well as activating FAO in order to support CSCs properties including self-renewal, tumorigenesis, and chemoresistance [27]. In addition, CSCs have higher lipid droplets (LDs) when compared with cancer cells. This is important for CSCs during metabolic stress because it sustains free fatty acids for ATP production via FAO and protects lipid peroxidation, a process producing lipid peroxides that can cause cell death [26]. In our study, we found that many lipid species, especially TGs, were upregulated in recurrence patients, suggesting that lipids are important for cancer cells to develop recurrence. To further investigate whether high levels of lipids in CCA recurrence are associated with the existence of CSCs, the expression of proteins involved in lipid metabolism and CSC markers was investigated. CD36 is known as a transmembrane glycoprotein and is expressed in various tumor types [55]. CD36 plays a critical role in cancer progression, including cancer proliferation and metastasis [56], and it is also associated with poor survival of cancer patients [57]. In CSCs, the elevated expression of CD36 was found and the uptake of an oxidized phospholipid, the ligand of CD36 drives glioma CSC proliferation, suggesting that the expression of CD36 is associated with CSC progression [30]. Similar to our finding that showed high expression of CD36, a protein involved in lipid uptake, was associated with a high expression of CSC markers. Moreover, the high level of CD36 was associated with lower recurrence-free survival, suggesting that high levels of lipid in recurrence patients may lead to high lipid uptake which benefits CSC survival and leads to CCA recurrence.

Reprogramming of metabolism is associated with cancer recurrence. Therefore, metabolomics-based biomarker discovery is widely used to discover biomarkers to predict cancer recurrence. In ovarian cancer, metabolic biomarkers showed the potential to predict recurrence with a high value of AUC [15, 58]. In good agreement with this report, we found that metabolic biomarkers have the potential to predict CCA recurrence, as evidenced by the high values of ACU, sensitivity, and specificity. Moreover, the association of potential metabolic biomarkers was further analyzed with respect to recurrence-free survival time. Our results showed that metabolic biomarkers have the potential to predict CCA recurrence. Taken together, our results hence highlight the important of metabolomics for reveals the molecular mechanisms of CCA recurrence and the potential biomarkers for the recurrence in early-stage cholangiocarcinoma.

## Conclusions

These findings reveal an alteration of the metabolic profile associated with recurrence. These metabolic changes may be associated with the existence of CSCs that lead to CCA recurrence. Moreover, the alteration of metabolites was shown to provide potential biomarkers for CCA recurrence. Therefore, the differential metabolites between patients with and without recurrence demonstrate, in the current exploratory study, the promising biomarker panel for CCA recurrence despite the larger cohort validation that remains to be elucidated.

## Supplementary Information

**Additional file 1: Fig. S1.** Kaplan-Meier curves representing the correlation between patient characteristics with recurrence-free survival. (A-H) The result from the different groups relating to age, gender, tumor site, histology type, primary (T) tumor stage, lymph node (N) metastasis status, distant metastasis (M) status, TNM stage. p-value lower than 0.05 was considered as a significantly value. **Fig. S2.** Kaplan-Meier curves representing the correlation between patient characteristics with overall survival. (A-H) The result from the different groups representing age, gender, tumor site, histology type, primary (T) tumor stage, lymph node (N) metastasis status, distant metastasis (M) status, TNM stage. *﻿p*-value lower than 0.05 was considered as a significantly value.

**Additional file 2: Table S1.** Patient characteristics on ^1^H-NMR analysis. **Table S2.** Patient characteristics on UPLC-MS analysis. **Table S3.** Total metabolites were identified in serum using ^1^H-NMR. **Table S4.** The differential lipid species of patients with and without recurrence. **Table S5.** The correlation of protein involved in lipid metabolism (CD36, ACLY, SCD1) and CSC markers (CD44, CD44v6, CD44v8-10, EpCAM)

## Data Availability

The datasets used and/or analyzed during the current study are available from the corresponding author on reasonable request.
