# Supplementary figures and images for: Integration of global metabolomics and lipidomics approaches reveals the molecular mechanisms and the potential biomarkers for postoperative recurrence in early-stage cholangiocarcinoma
Source: Cancer Metab. 2021 Aug 4;9:30. doi: 10.1186/s40170-021-00266-5 (PMC8335966; doi:10.1186/s40170-021-00266-5)

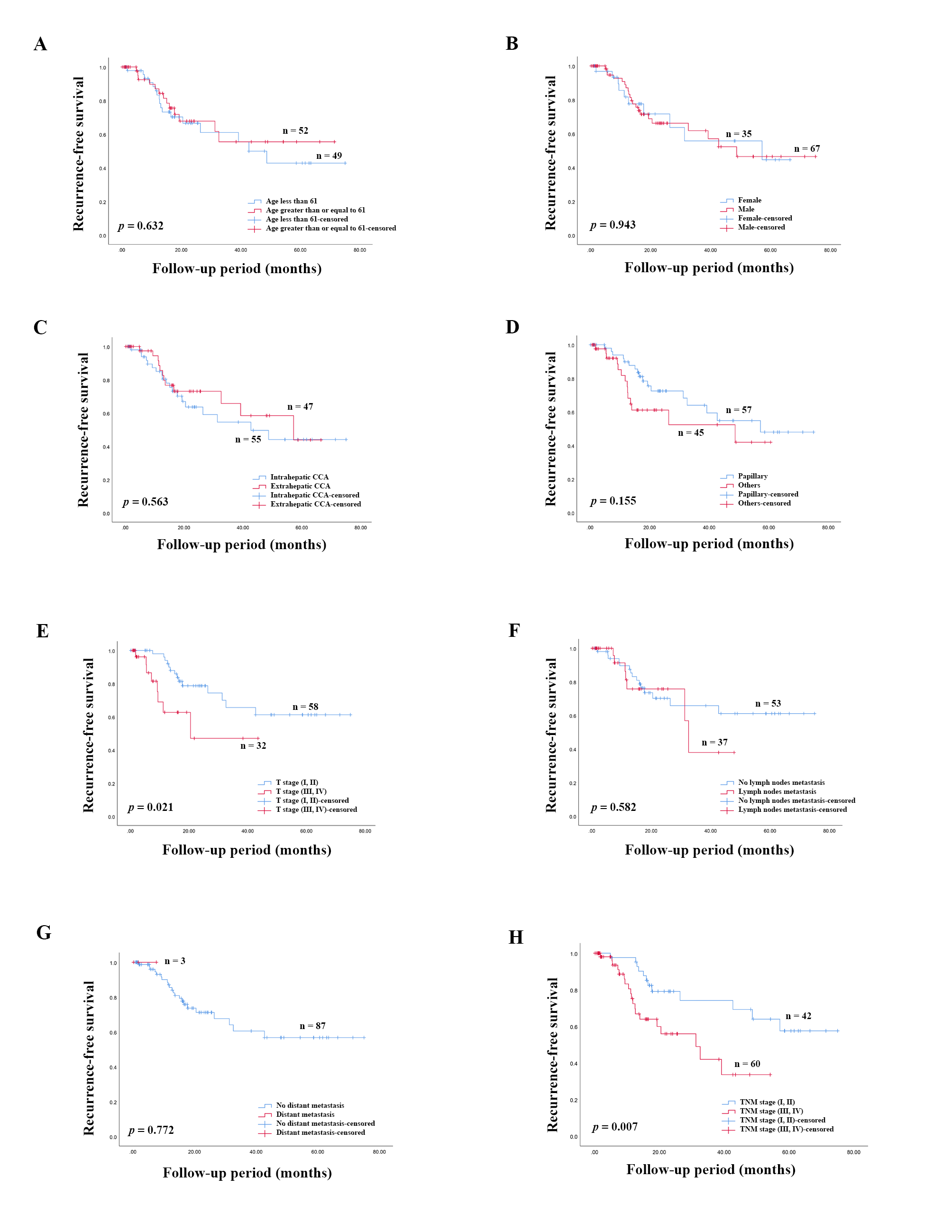

Supplement: Supplementary file 1 — Additional file 1: Fig. S1. Kaplan-Meier curves representing the correlation between patient characteristics with recurrence-free survival. (A-H) The result from the different groups relating to age, gender, tumor site, histology type, primary (T) tumor stage, lymph node (N) metastasis status, distant metastasis (M) status, TNM stage. p-value lower than 0.05 was considered as a significantly value. Fig. S2. Kaplan-Meier curves representing the correlation between patient characteristics with overall survival. (A-H) The result from the different groups representing age, gender, tumor site, histology type, primary (T) tumor stage, lymph node (N) metastasis status, distant metastasis (M) status, TNM stage. ﻿p-value lower than 0.05 was considered as a significantly value. [file 40170_2021_266_MOESM1_ESM.zip › Additional file 2. Fig. S1.tif]

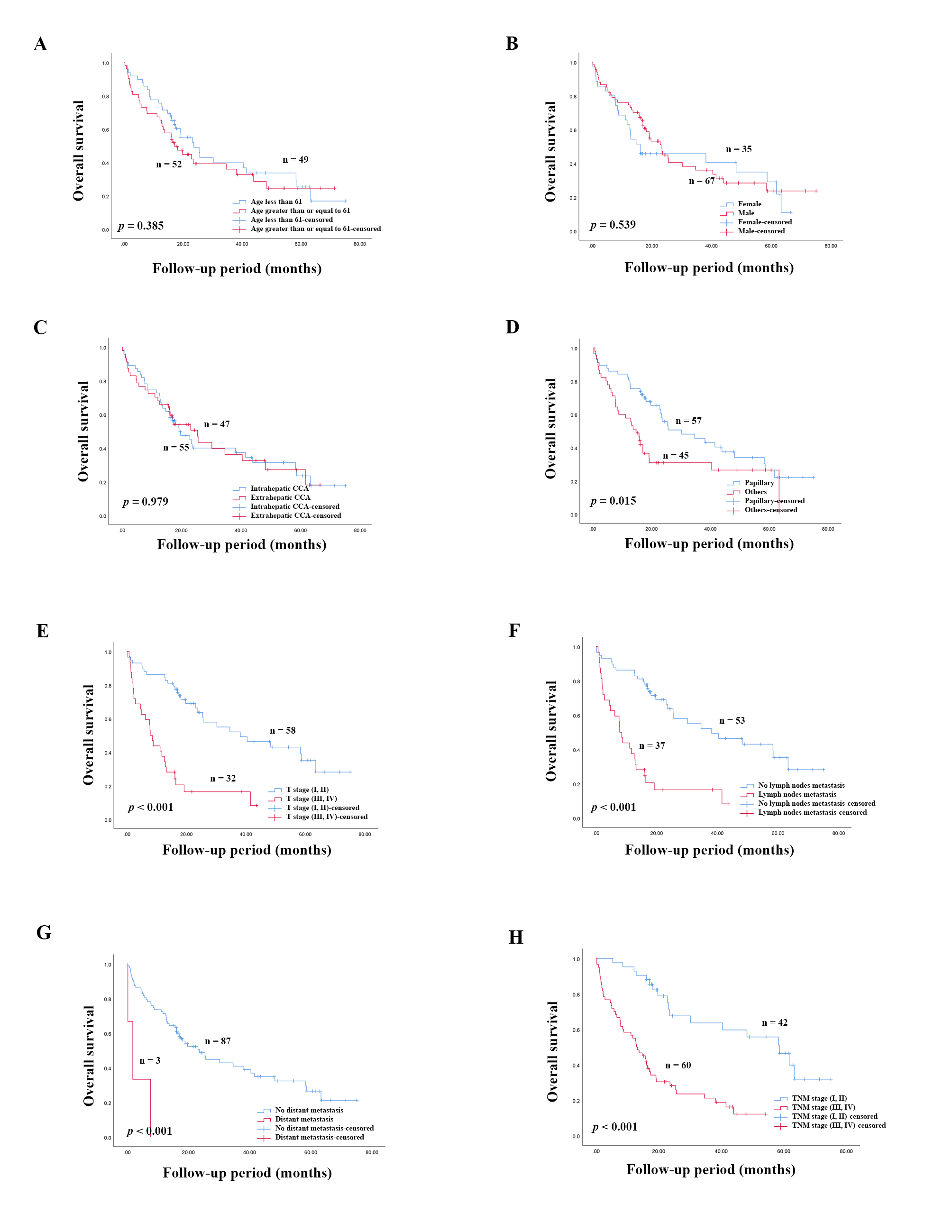

Supplement: Supplementary file 1 — Additional file 1: Fig. S1. Kaplan-Meier curves representing the correlation between patient characteristics with recurrence-free survival. (A-H) The result from the different groups relating to age, gender, tumor site, histology type, primary (T) tumor stage, lymph node (N) metastasis status, distant metastasis (M) status, TNM stage. p-value lower than 0.05 was considered as a significantly value. Fig. S2. Kaplan-Meier curves representing the correlation between patient characteristics with overall survival. (A-H) The result from the different groups representing age, gender, tumor site, histology type, primary (T) tumor stage, lymph node (N) metastasis status, distant metastasis (M) status, TNM stage. ﻿p-value lower than 0.05 was considered as a significantly value. [file 40170_2021_266_MOESM1_ESM.zip › Additional file 2. Fig. S2.tif]
