# Supplementary material for: Integration of global metabolomics and lipidomics approaches reveals the molecular mechanisms and the potential biomarkers for postoperative recurrence in early-stage cholangiocarcinoma
Source: Cancer Metab. 2021 Aug 4;9:30. doi: 10.1186/s40170-021-00266-5 (PMC8335966; doi:10.1186/s40170-021-00266-5)
Supplement: Supplementary file 2 — Additional file 2: Table S1. Patient characteristics on 1H-NMR analysis. Table S2. Patient characteristics on UPLC-MS analysis. Table S3. Total metabolites were identified in serum using 1H-NMR. Table S4. The differential lipid species of patients with and without recurrence. Table S5. The correlation of protein involved in lipid metabolism (CD36, ACLY, SCD1) and CSC markers (CD44, CD44v6, CD44v8-10, EpCAM) [file 40170_2021_266_MOESM2_ESM.docx]

**Table S1.** Patient characteristics on ^1^H-NMR analysis.

| **Characteristics** | **Early stage (n=36)** | | **Late stage (n=55)** | |
| --- | --- | --- | --- | --- |
|  | **NR (n=26)** | **R (n=10)** | **NR (n=37)** | **R (n=18)** |
|  |  |  |  |  |
| Median age (rage) | 61 (49-70) | 59 (43-73) | 62 (44-82) | 61 (53-71) |
| Sex  Female  Male | 10  16 | 3  7 | 11  26 | 7  11 |
| Tumor site  Intrahepatic  Extrahepatic | 17  9 | 5  5 | 15  22 | 13  5 |
| Histology type  Papillary  Others | 23  3 | 7  3 | 15  22 | 9  9 |
| Primary tumor (T)  I, II  III, IV  NA | 23  1  2 | 7  0  3 | 15  21  1 | 4  8  6 |
| Lymph nodes (N) metastasis  No  Yes  NA | 23  1  2 | 7  0  3 | 10  26  1 | 6  6  6 |
| Distant metastasis (M)  No  Yes  NA | 24  0  2 | 7  0  3 | 34  2  1 | 12  0  6 |
| NR: non-recurrence, R: recurrence, NA: not applicable | | | | |

**Table S2.** Patient characteristics on UPLC-MS analysis.

| **Characteristics** | **Early stage (n=42)** | | **Late stage (n=59)** | |
| --- | --- | --- | --- | --- |
|  | **NR (n=30)** | **R (n=12)** | **NR (n=40)** | **R (n=19)** |
|  |  |  |  |  |
| Median age (rage) | 61 (49-74) | 59 (43-73) | 61 (44-79) | 60 (53-71) |
| Sex  Female  Male | 12  18 | 3  9 | 13  27 | 7  12 |
| Tumor site  Intrahepatic  Extrahepatic | 18  12 | 6  6 | 17  23 | 13  6 |
| Histology type  Papillary  Others | 23  7 | 8  4 | 17  23 | 9  10 |
| Primary tumor (T)  I, II  III, IV  NA | 27  1  2 | 9  0  3 | 17  22  1 | 5  8  6 |
| Lymph nodes (N) metastasis  No  Yes  NA | 27  1  2 | 9  0  3 | 10  29  1 | 6  7  6 |
| Distant metastasis (M)  No  Yes  NA | 28  0  2 | 9  0  3 | 36  3  1 | 13  0  6 |
| NR: non-recurrence, R: recurrence, NA: not applicable | | | | |

**Table S3.** Total metabolites were identified in serum using ^1^H-NMR.

| **Metabolites** | **Early stage** | | | **Late stage** | | |
| --- | --- | --- | --- | --- | --- | --- |
|  | **Concentration (mM)** | | ***p*-value** | **Concentration (mM)** | | ***p*-value** |
|  | **NR** | **R** |  | **NR** | **R** |  |
| Leucine | 0.71(0.53) | 0.45(0.55) | **0.031** | 0.71(0.60) | 0.71(0.32) | 0.584 |
| Valine | 0.58(0.39) | 0.40(0.38) | **0.028** | 0.54(0.38) | 0.55(0.20) | 0.971 |
| Isoleucine | 0.95(0.53) | 0.61(0.70) | **0.041** | 0.89(0.58) | 0.92(0.30) | 0.823 |
| β-aminoisobutyrate | 0.74(0.27) | 0.55(0.39) | 0.109 | 0.63(0.31) | 0.73(0.25) | 0.226 |
| β-hydroxybutyrate | 0.82(0.44) | 0.61(0.35) | 0.271 | 0.73(0.37) | 0.79(0.39) | 0.900 |
| Lactate | 10.01(7.38) | 7.12(4.23) | 0.053 | 10.43(9.84) | 10.42(7.07) | 0.590 |
| Alanine | 1.16(0.95) | 0.96(0.69) | 0.101 | 1.17(0.80) | 1.20(0.45) | 0.788 |
| Arginine | 1.03(0.74) | 0.67(0.74) | **0.045** | 1.01(0.81) | 1.04(0.43) | 0.615 |
| Acetate | 1.38(0.91) | 0.92(0.58) | 0.053 | 1.41(1.09) | 1.34(1.34) | 0.473 |
| Proline | 5.05(2.33) | 3.78(3.04) | 0.101 | 4.72(1.91) | 5.10(2.46) | 0.566 |
| Acetoacetate | 0.55(0.43) | 0.40(0.21) | 0.058 | 0.50(0.40) | 0.47(0.25) | 0.240 |
| Glutamate | 1.41(0.63) | 0.97(0.68) | **0.049** | 1.37(0.64) | 1.39(0.73) | 0.830 |
| Pyruvate | 0.43(0.24) | 0.26(0.27) | **0.049** | 0.43(0.29) | 0.39(0.26) | 0.572 |
| Succinate | 0.15(0.09) | 0.11(0.09) | **0.031** | 0.15(0.11) | 0.14(0.09) | 0.420 |
| Citrate | 0.38(0.20) | 0.28(0.21) | **0.012** | 0.38(0.15) | 0.40(0.12) | 0.893 |
| NR: non-recurrence, R: recurrence, concentration reported as median with interquartile range (IQR) | | | | | | |

**Table S3.** Total metabolites were identified in serum using ^1^H-NMR (Cont.).

| **Metabolites** | **Early stage** | | | **Late stage** | | |
| --- | --- | --- | --- | --- | --- | --- |
|  | **Concentration (mM)** | | ***p*-value** | **Concentration (mM)** | | ***p*-value** |
|  | **NR** | **R** |  | **NR** | **R** |  |
| Dimethylamine | 0.10(0.05) | 0.07(0.05) | **0.034** | 0.08(0.05) | 0.09(0.04) | 0.865 |
| Sarcosine | 0.25(0.16) | 0.16(0.09) | **0.007** | 0.23(0.18) | 0.27(0.10) | 0.513 |
| p-Hydroxyphenyllacate | 0.20(0.13) | 0.14(0.09) | 0.063 | 0.17(0.07) | 0.18(0.08) | 0.733 |
| Creatine | 0.57(0.34) | 0.37(0.35) | **0.031** | 0.49(0.32) | 0.54(0.17) | 0.914 |
| Creatinine | 0.51(0.33) | 0.35(0.30) | **0.041** | 0.46(0.35) | 0.49(0.20) | 0.914 |
| Phosphocreatine | 0.23(0.16) | 0.16(0.14) | **0.026** | 0.22(0.19) | 0.25(0.09) | 0.943 |
| Choline | 0.74(0.49) | 0.53(0.41) | **0.049** | 0.51(0.41) | 0.55(0.26) | 0.964 |
| Glycine | 0.82(0.36) | 0.74(0.49) | 0.303 | 0.82(0.37) | 0.88(0.30) | 0.693 |
| Glucose | 1.21(1.13) | 0.85(0.41) | **0.037** | 0.82(0.79) | 1.03(0.66) | 0.370 |
| Tyrosine | 0.20(0.10) | 0.16(0.14) | 0.189 | 0.20(0.15) | 0.20(0.11) | 0.706 |
| Histidine | 0.54(0.31) | 0.40(0.37) | 0.068 | 0.51(0.28) | 0.51(0.17) | 0.844 |
| Phenylalanine | 0.66(0.26) | 0.45(0.54) | 0.201 | 0.66(0.48) | 0.71(0.27) | 0.788 |
| Adenine | 0.15(0.14) | 0.12(0.12) | 0.214 | 0.16(0.16) | 0.11(0.17) | 0.203 |
| Formate | 0.13(0.48) | 0.09(0.08) | **0.021** | 0.12(0.08) | 0.13(0.09) | 0.816 |
| NR: non-recurrence, R: recurrence, concentration reported as median with interquartile range (IQR) | | | | | | |

**Table S4.** The differential lipid species of patients with and without recurrence.

| **Metabolites** | **m/z** | **rt (min)** | **ppm** | **LoA** | **m/z theoretical** | **Adduct** | **Mode** | ***p*-value** | **FDR** | **VIP** |
| --- | --- | --- | --- | --- | --- | --- | --- | --- | --- | --- |
| PC(P-18:0/22:6) | 818.6057 | 6.49 | 0 | 1 | 818.6058 | [M+H]^+^ | pos | 0.00369 | 0.045 | 1.00 |
| PE(20:3/18:1) | 768.5567 | 7.00 | 3 | 3 | 768.5538 | [M+H]^+^ | pos | 0.00220 | 0.035 | 1.40 |
| DG(20:0/18:2) | 685.5535 | 9.04 | 30 | 1 | 685.5741 | [M+Na]^+^ | pos | 0.00114 | 0.025 | 2.27 |
| DG(18:1/22:5) | 686.5568 | 9.04 | 22 | 1 | 686.5718 | [M+NH_4_]^+^ | pos | 0.00080 | 0.022 | 1.62 |
| TG(14:0/15:0/24:0) | 899.7917 | 10.64 | 13 | 1 | 899.8038 | [M+Na]^+^ | pos | 0.00198 | 0.034 | 1.60 |
| TG(18:1/16:1/20:3) | 898.7886 | 10.64 | 3 | 1 | 898.7858 | [M+NH_4_]^+^ | pos | 0.00220 | 0.035 | 2.05 |
| TG(16:1/18:1/20:3) | 903.7435 | 10.64 | 3 | 1 | 903.7412 | [M+Na]^+^ | pos | 0.00245 | 0.036 | 1.06 |
| TG(18:1/18:1/18:2) | 900.8046 | 10.76 | 3 | 3 | 900.8015 | [M+NH_4_]^+^ | pos | 0.00026 | 0.015 | 3.28 |
| TG(15:0/18:0/22:2) | 901.8079 | 10.76 | 15 | 1 | 901.8219 | [M+H]^+^ | pos | 0.00013 | 0.014 | 2.61 |
| TG(18:1/18:0/18:2) | 902.8106 | 10.76 | 7 | 3 | 902.8171 | [M+NH_4_]^+^ | pos | 0.00019 | 0.015 | 1.48 |
| TG(18:0/18:2/18:2) | 905.7598 | 10.76 | 3 | 3 | 905.7569 | [M+Na]^+^ | pos | 0.00004 | 0.014 | 1.60 |
| TG(15:0/18:3/22:5) | 906.7628 | 10.76 | 9 | 1 | 906.7545 | [M+NH_4_]^+^ | pos | 0.00008 | 0.014 | 1.21 |
| TG(18:1/20:1/18:3) | 926.8198 | 10.85 | 3 | 1 | 926.8171 | [M+NH_4_]^+^ | pos | 0.00301 | 0.040 | 1.17 |
| TG(15:0/22:6/O-18:0) | 879.8136 | 10.98 | 38 | 1 | 879.7800 | [M+H]^+^ | pos | 0.00159 | 0.030 | 1.21 |
| TG(18:0/16:0/18:1) | 878.8100 | 10.98 | 8 | 3 | 878.8171 | [M+NH_4_]^+^ | pos | 0.00178 | 0.032 | 2.89 |
| TG(18:1/16:0/18:1) | 876.8044 | 10.98 | 3 | 3 | 876.8015 | [M+NH_4_]^+^ | pos | 0.00220 | 0.035 | 6.60 |
| m/z: mass to charge ratio, rt: retention time, ppm: part per million, LoA: levels of analysis, FDR: false discovery rate,  VIP: variable importance in projection, DG: driacylglycerol, TG: triacylglycerol, PC: phosphatidylcholine,  PA: phosphatidic acid, PE: phosphatidylethanolamine, pos: positive mode, neg: negative mode | | | | | | | | | | |

**Table S4.** The differential lipid species of patients with and without recurrence (Cont.).

| **Metabolites** | **m/z** | **rt (min)** | **ppm** | **LoA** | **m/z theoretical** | **Adduct** | **Mode** | ***p*-value** | **FDR** | **VIP** |
| --- | --- | --- | --- | --- | --- | --- | --- | --- | --- | --- |
| TG(15:0/16:0/22:0) | 877.8075 | 10.98 | 16 | 1 | 877.8219 | [M+H]^+^ | pos | 0.00198 | 0.034 | 5.07 |
| TG(18:1/18:0/18:1) | 904.8265 | 11.01 | 6 | 3 | 904.8328 | [M+NH_4_]^+^ | pos | 0.00022 | 0.015 | 2.06 |
| TG(16:0/20:2/18:1) | 902.8204 | 11.01 | 4 | 3 | 902.8171 | [M+NH_4_]^+^ | pos | 0.0002 | 0.015 | 4.42 |
| TG(15:0/22:0/18:1) | 903.8236 | 11.01 | 15 | 1 | 903.8375 | [M+H]^+^ | pos | 0.00029 | 0.015 | 3.51 |
| TG(14:0/20:1/20:2) | 907.7756 | 11.01 | 3 | 1 | 907.7725 | [M+Na]^+^ | pos | 0.00010 | 0.014 | 2.05 |
| TG(15:0/20:3/20:4) | 908.7788 | 11.01 | 10 | 1 | 908.7702 | [M+NH_4_]^+^ | pos | 0.00008 | 0.014 | 1.57 |
| TG(15:0/20:1/20:5) | 910.7943 | 11.24 | 9 | 1 | 910.7858 | [M+NH_4_]^+^ | pos | 0.00090 | 0.022 | 1.05 |
| TG(20:0/15:0/20:0) | 905.8393 | 11.24 | 15 | 1 | 905.8532 | [M+H]^+^ | pos | 0.00090 | 0.022 | 1.85 |
| TG(18:0/18:0/18:1) | 906.8419 | 11.24 | 7 | 3 | 906.8484 | [M+NH_4_]^+^ | pos | 0.00080 | 0.022 | 1.06 |
| TG(18:0/16:0/20:2) | 904.8359 | 11.24 | 3 | 3 | 904.8328 | [M+NH_4_]^+^ | pos | 0.00080 | 0.022 | 2.36 |
| PA(21:0/18:3) | 739.5109 | 6.32 | 23 | 1 | 739.5283 | [M-H]^-^ | neg | 0.00033 | 0.031 | 1.26 |
| PE(16:0/20:4) | 738.5079 | 6.32 | 0 | 1 | 738.5079 | [M-H]^-^ | neg | 0.00033 | 0.031 | 1.87 |
| PE(18:0/16:1) | 716.5234 | 6.86 | 0 | 1 | 716.5236 | [M-H]^-^ | neg | 0.00063 | 0.033 | 1.14 |
| PA(22:1/19:2) | 767.5426 | 7.01 | 22 | 1 | 767.5596 | [M-H]^-^ | neg | 0.00056 | 0.031 | 1.69 |
| PE(18:1/20:3) | 766.5395 | 7.01 | 0 | 1 | 766.5392 | [M-H]^-^ | neg | 0.00043 | 0.031 | 2.49 |
| PE(18:1/18:1) | 742.5392 | 7.03 | 0 | 1 | 742.5392 | [M-H]^-^ | neg | 0.00043 | 0.031 | 1.32 |
| m/z: mass to charge ratio, rt: retention time, ppm: part per million, LoA: levels of analysis, FDR: false discovery rate,  VIP: variable importance in projection, DG: driacylglycerol, TG: triacylglycerol, PC: phosphatidylcholine,  PA: phosphatidic acid, PE: phosphatidylethanolamine, pos: positive mode, neg: negative mode | | | | | | | | | | |

**Table S5.** The correlation of protein involved in lipid metabolism (CD36, ACLY, SCD1) and CSC markers (CD44, CD44v6, CD44v8-10, EpCAM).

|  |  | **ACLY** | **SCD1** | **CD44** | **CD44v6** | **CD44v8-10** | **EpCAM** |
| --- | --- | --- | --- | --- | --- | --- | --- |
| **CD36** | Pearson Correlation | -0.041 | -0.001 | 0.306 | 0.405 | 0.369 | 0.211 |
|  | *p*-value | 0.733 | 0.995 | **0.009** | **<0.001** | **0.001** | 0.074 |
| **ACLY** | Pearson Correlation | 1 | 0.327 | 0.023 | -0.154 | -0.067 | 0.095 |
|  | *p*-value |  | **0.005** | 0.846 | 0.193 | 0.573 | 0.424 |
| **SCD1** | Pearson Correlation |  | 1 | 0.033 | -0.088 | 0.016 | -0.075 |
|  | *p*-value |  |  | 0.779 | 0.462 | 0.893 | 0.526 |
| **CD44** | Pearson Correlation |  |  | 1 | 0.164 | 0.266 | -0.026 |
|  | *p*-value |  |  |  | 0.166 | **0.023** | 0.829 |
| **CD44v6** | Pearson Correlation |  |  |  | 1 | 0.453 | 0.307 |
|  | *p*-value |  |  |  |  | **<0.001** | **0.008** |
| **CD44v8-10** | Pearson Correlation |  |  |  |  | 1 | 0.143 |
|  | *p*-value |  |  |  |  |  | 0.226 |
| CD: cluster of differentiation, ACLY: ATP citrate lyase, SCD1: stearoyl-CoA desaturase-1  EpCAM: epithelial cell adhesion molecule | | | | | | | |
